# A scoping review of historical and contemporary management strategies for paediatric scrotal trauma

**DOI:** 10.1007/s11845-025-03929-0

**Published:** 2025-03-15

**Authors:** Kenneth Patterson, Derek B. Hennessey, Fardod O’Kelly

**Affiliations:** 1 Department of Urology, Tallaght University Hospital, Dublin, Ireland; 2 Department of Urology, Mercy University Hospital, Cork, Ireland; 3 Department of Paediatric Urology, Beacon Hospital, Dublin, Ireland; 4 Department of Surgery, University College Dublin, Dublin, Ireland

**Keywords:** Delayed repair, Scrotal trauma, Scrotal ultrasound, Testicular atrophy, Testicular fracture

## Abstract

**Objective:**

To perform a scoping review of the literature pertaining to paediatric scrotal trauma and to contrast operative with conservative management in this cohort of patients using available data.

**Methods:**

A search of Cochrane, SCOPUS, and EMBASE databases was performed using methods pre-published on PROSPERO. Reporting followed Preferred Reporting Items for Systematic Review and Meta-analysis guidelines. Eligible studies were articles or abstracts published in English describing the management of paediatric scrotal trauma, which reported at least one secondary outcome. The intended primary analysis is to report the management of paediatric scrotal trauma and the outcome based on management.

**Results:**

Thirty-six studies were identified, totaling 253 patients. Then, 91.7% of cases presented with unilateral testicular injury and 94.5% of cases resulted from blunt trauma. Then, 86% of patients presenting with scrotal trauma underwent ultrasound imaging of the scrotum. One hundred twenty-three cases underwent conservative management, 116 cases underwent acute surgical management, and 14 underwent delayed surgical management, with a mean time to an intervention of 3 days. Thirty patients were found to have testicular atrophy, with a mean follow-up of 14 months, of these 30 patients, 63% (*n* = 19) were conservatively managed, 20% (*n* = 6) were managed with acute surgical repair, and 17% (*n* = 5) were managed with delayed surgical repair.

**Conclusion:**

Paediatric testicular trauma is a rare presentation. A high level of suspicion is mandatory when testicular rupture is suspected. Early exploration is warranted in the setting of high risk and provides an excellent chance of testicular salvage.

## Introduction

Blunt scrotal trauma in paediatric patients is a rare phenomenon, but one which requires prompt identification, diagnosis, and decision on management. Among all forms of trauma-related injuries, scrotal trauma accounts for just 1%, due to factors such as the anatomic location of the scrotum, the protective effect of the fluid between the parietal and visceral layers of the tunica vaginalis, and the mobility of the testes within the scrotum [1, 2]. The most common mechanism of scrotal injury is blunt trauma, which most commonly results from a sports injury (projectile, crush, and kick), road traffic accidents, straddle injury from bikes, and assaults [3]. The resulting injury is often the consequence of the testis becoming compressed between the groin or the pubic symphysis.

The manifestations of testicular injury from scrotal trauma are varied and can include testicular fracture, testicular rupture, testicular torsion, intratesticular haemorrhage/haematoma, and in rare cases, testicular dislocation [4]. Testicular fracture is the result of a rip/tear in the tunica albuginea of the testis, an injury which is present in approximately 50% of cases presenting with a traumatic haematocele [3, 4]. Clinical presentation of scrotal trauma is with pain, acute onset swelling, bruising, and scrotal skin laceration. Clinical examination of paediatric patients with scrotal trauma can be very challenging due to the pain associated with the examination of the area and the emotional anxiety surrounding injury to the genital area by both patient and parents, both of which can make prompt diagnosis even more important in these patients.

Ultrasound imaging with colour Doppler visualisation can be very beneficial in the rapid diagnosis of these patients for this reason of difficulty in clinical examination. Ultrasound with colour flow and duplex Doppler is an imaging modality that can characterise the different manifestations of scrotal trauma to a high degree of accuracy in the hands of an experienced radiologist. Scrotal ultrasound can be utilised in children using a linear array flow transducer with a frequency of between 5 and 10 mHz [1]. This is a relatively quick and painless method of establishing what type of injury may be present, without subjecting the child to a clinical examination which can be both painful and distressing for them.

The European Association of Urology (EAU) and American Urology Association (AUA) guidelines on urogenital trauma still recommend that patients with suspected testicular rupture should undergo urgent scrotal exploration with the closure of tunica albuginea or orchidectomy in non-salvageable cases [5, 6]. The rationale for early exploration and fracture repair is to preserve future fertility, reduce the risk of delayed orchidectomy, and prevent future testicular atrophy. However, the management of these cases in children is disparate with some groups electing to perform early surgical repair, some proposing delayed repair (> 24–48 h), and some preferring to manage this conservatively. Furthermore, there are no consensus treatments as to the optimal management algorithm [2, 7, 8].

The aim of our study was to perform a scoping review of the current literature on paediatric scrotal trauma in order to identify the number of patients, the type of intervention offered to these patients, to compare an operative with a conservatively managed cohort of these patients, and to assess what outcomes data exists in terms of follow-up, post-operative complications, and testicular outcomes with respect to atrophy.

## Methods

This scoping review was registered with the international prospective register of systematic reviews (PROSPERO) and assigned the registration CRD42019143463. Institutional research and ethics board review was waived.

We used the PRISMA (Preferred Reporting Items for Systematic Reviews and Meta-Analyses) guidelines to formulate the basis of pre-specified eligibility criteria using the PICO (P—Populations/People/Patient/Problem, I—Intervention(s), C—Comparison, O—Outcome) worksheet and search strategy. A systematic search of the English literature was performed on 1st May 2020 to identify systematic reviews and meta-analyses focusing on clinical paediatric urology from 1st January 1946 to 30th April 2020. An electronic search was performed using PubMed, Scopus, and Embase databases. Boolean search terms were limited to variations of “testis injury” and/or “testicular trauma” and/or “pediatric.” We had the aid of a librarian with a masters in librarian studies to assist with the Medline/Embase search (Appendix 1).

Only children were included in the results of this review. Exclusion criteria were as follows: adult only papers (patients > 18 years old), papers that included a mix of adult and paediatric patients but did not distinguish between the two cohorts, editorials, surveys, letters to the editor, book chapters, conference proceedings, non-urological papers, and those which were basic science research (non-clinical). Following identification, the records were screened by two (KP and DH) independent reviewers based on title and abstract. Those that met inclusion criteria were screened again through a full-text review. Following the full-text review, the final included records were identified. The data from the included studies were summarised. Interobserver reliability was assessed using the kappa statistic with + 1 indicating perfect agreement, and − 1 indicating complete disagreement. Any disputes were presented to a third author (FOK) for consensus resolution.

Risk of bias was assessed using the Newcastle–Ottawa Scale for evaluating the quality of the non-randomised studies relating to paediatric blunt testicular trauma outcomes. Three factors were considered to score the quality of included studies: (1) selection, including representativeness of the exposed cohort, selection of the non-exposed cohort, ascertainment of exposure, and demonstration that at the start of the study the outcome of interest was not present; (2) comparability, assessed on the basis of study design and analysis, and whether any confounding variables were adjusted for; and (3) outcome, based on the follow-up period and cohort retention [9] (Appendix 2). To evaluate the strength of evidence from the included data, the Grading of Recommendation Assessment, Development, and Evaluation (GRADE) method will be used [9].

An electronic database was created in which study type, author name, publication date, journal, topic, and study type (case series, retrospective study, etc.), where possible perioperative and post-operative outcomes were captured. Differences in means between groups were measured, and risk ratios applied where applicable. Univariate analysis was performed for each variable, with a *p*-value of < 0.05 considered statistically significant. Statistical analyses were performed on Prism statistical software (GraphPad v6.0; California).

## Results

**Fig. 1:**
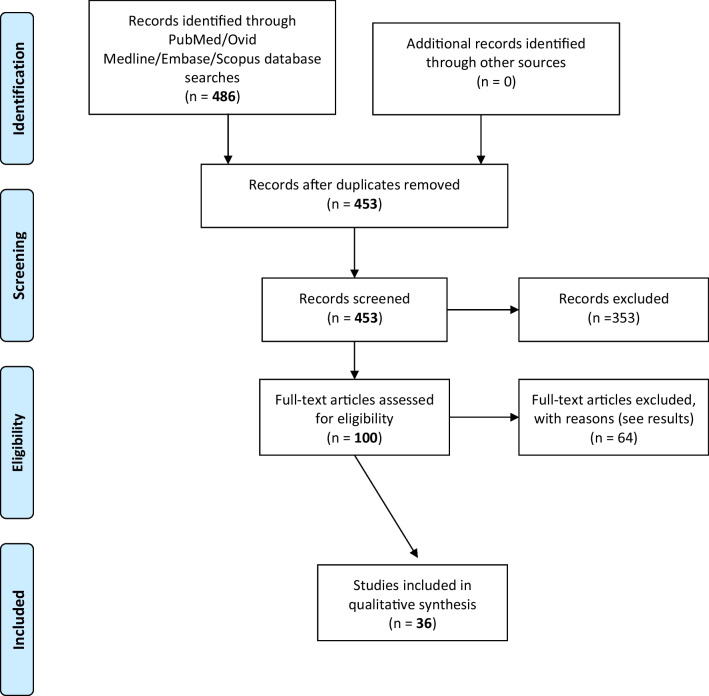
Assessment of eligibility of paediatric scrotal trauma manuscripts for study review

Database searches returned 486 results, with no additional material was identified from a search of grey literature. Following the removal of duplicate results, 453 articles moved into title and abstract screening. Following the exclusion of irrelevant articles based on title and abstract, including studies that included a mix of paediatric and adult patients, 100 full-text articles were assessed through full-text review. Articles were excluded if the full text was not available at the time of search (twenty-eight articles), if they were review articles with no patients with testicular injuries (fourteen articles), if there were no details on the type of testicular injury or treatment used for injury (nine articles), if the full text was not available in English (seven articles), if there were no paediatric patients involved in the study (four articles), and if the study did not involve human subjects (two studies). In total, 36 studies were found to fulfil eligibility criteria and were included in our study (Fig. 1). Interobserver reliability was found to be 0.9, suggesting a high level of agreement. The overall GRADE level of evidence was low. The majority of these studies were observational studies which contained important inconsistencies such as a lack of outcomes data, sparse data, and a high probability of reporting bias.

**Fig. 2:**
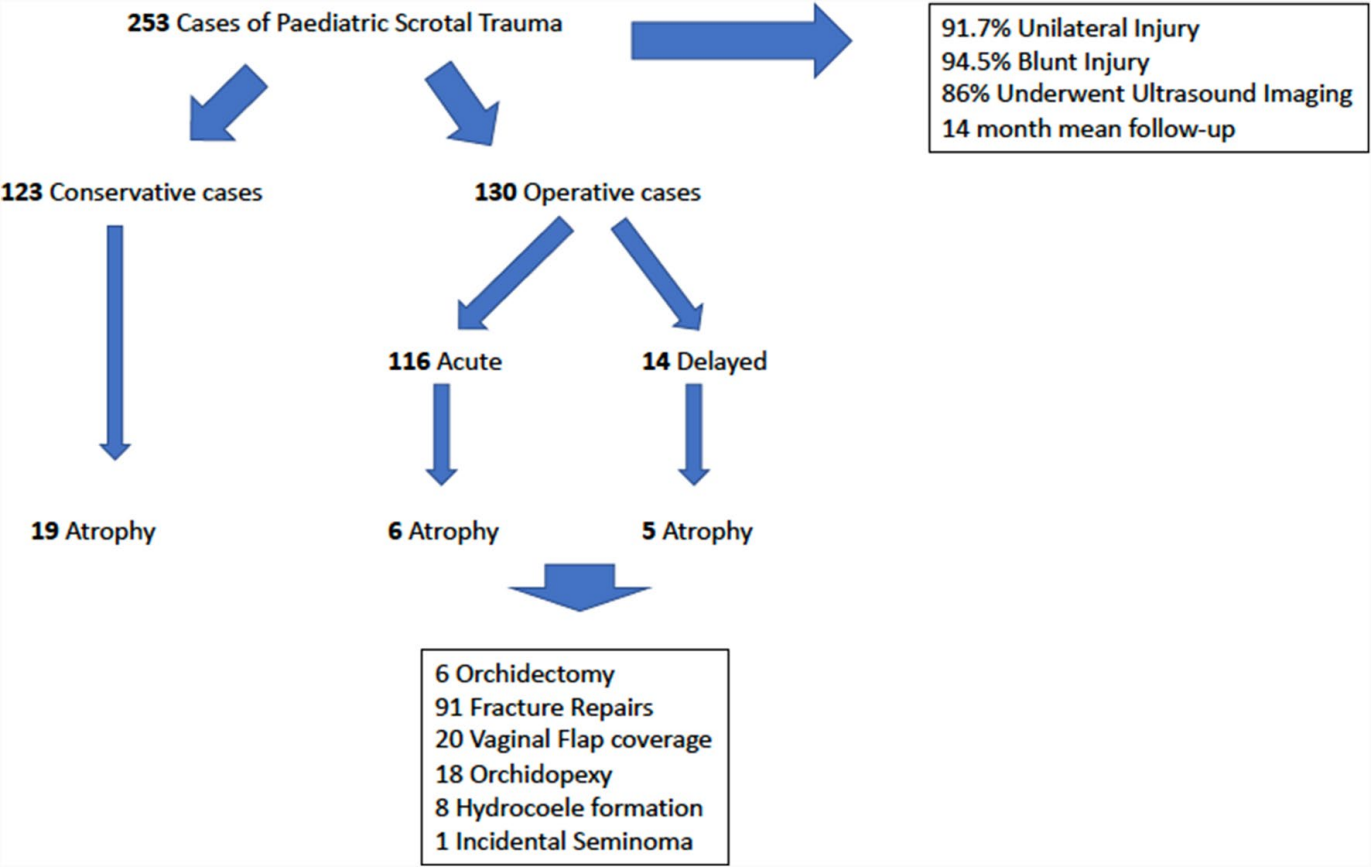
Note: This data is mandatory. Please provide missing figure caption

These studies originated from 15 different countries, were published between the years of 1969–2020, and included 253 unique paediatric patients with scrotal trauma. Figure 2 represents a summary of the results of the 253 cases based on treatment modality. Then, 91.7% of cases presented with unilateral testicular injury, with 94.5% of cases resulting from blunt traumatic scrotal injury. Then, 86% of patients presenting with scrotal trauma underwent ultrasound imaging of the scrotum. One hundred twenty-three cases underwent conservative management, 116 cases underwent acute surgical management, and 14 underwent delayed surgical management, with a mean time to intervention of 3 days. Testicular atrophy was defined as either a significant difference in volume on subsequent ultrasound, a clinical reduction in the volume of the testis by about 50% compared to the contralateral side. Thirty patients (*n* = 30) were found to have testicular atrophy, with a mean follow-up of 14 months; of these 30 patients, 63% (*n* = 19) were conservatively managed, 20% (*n* = 6) were managed with acute surgical repair, and 17% (*n* = 5) were managed with delayed surgical repair (Table 1). Not all studies in this review were suitable for risk of bias assessment using the Newcastle–Ottawa tool, and therefore, only non-randomised observational studies with specifically mentioned outcomes were selected for assessment. This yielded nine studies available for comparison. Within these, a significant level of bias was ascertained with two studies receiving 3 out of a possible seven stars, and seven studies receiving 4 out of a possible seven stars (Table 1).

Pathological/operative findings of 144 testes were classified in the included studies, six patients underwent orchidectomy, 91 fracture repairs were performed, 20 vaginal flap covers, 18 orchidopexy, 8 traumatic hydrocele, and 1 incidental seminoma were discovered at operation. All surgically managed patients that have been included in our study had a confirmed breach in the tunica albuginea intraoperatively. Mechanism of injury had a significant effect on development of testicular atrophy, 13 patients with blunt testicular trauma following sporting injury developed testicular atrophy (*p* = 0.03) with 30% (*n* = 4) of these managed with acute or delayed surgical management and 69% (*n* = 9) managed with conservative management.

**Table 1:** Effect of approach, ultrasonography, and mechanism of injury in a paediatric blunt scrotal trauma population

Approach	Acute surgical	Delayed surgical	Conservative	*p*-value
Number of studies (*n)*	22	5	9	
Number of subjects (*n*)	116	14	123	
Mean time to intervention (days)	0.23	3.0	n/a	
Reported testicular atrophy, *n* (%)	6 (5.2)	5 (35.7)	19 (15.5)	**0.002**
Testicular fracture suspected on ultrasound imaging fraction (%)	21/80 (26.3)	19/42 (45.2)		**0.04**
Mechanism of injury leading to testicular atrophy				
Sporting, *n* (%)	4 (36.4)		10 (47.4)	**0.03**
Intentional/assault, *n* (%)	5 (45.5)		2 (10.5)	0.19
Not specified, *n* (%)	2 (18.2)		7 (26.8)	n/a

## Discussion

To our knowledge, this is the first comprehensive assessment of paediatric scrotal trauma, management, and outcomes performed. Given that the paediatric male population ranges in age from 0 to 17, there is a wide variety of activities and potential aetiologies of injuries amongst this age range. A high propensity of these injuries is attributable to sport [10]. With the ever-increasing popularity of high impact sports such as football, rugby, Gaelic games, and mixed martial arts (MMA), the likelihood is that these injuries may become more prevalent in the near future. For this reason, it is important to ensure there is clear guidance for urologists as to the diagnostics used, method of treatment, and likely outcomes for each, that can inform their decision-making.

As previously mentioned, physical examination can be very difficult in this cohort of young patients due to the emotional stress involved in such an injury and due to pain; for this reason, we postulate that physical examination alone is an unreliable means of definitively diagnosing testicular rupture, and in cases of clinical uncertainty, ultrasonography is a very useful adjunct to help confirm this diagnosis. Ultrasonography alone cannot replace the need for physical examination, however, as ultrasound can miss the presence of testicular fracture, particularly in the acute period following testicular rupture, as demonstrated in our figures in Table 1. Although there was no classification of severity of injury suggested in any of the included studies, we suggest that a severe injury be described as a disruption in the *Tunica albuginea* with either loss of testicular homogeneity or an alteration to the internal testicular architecture (e.g., intratesticular haematoma and/or loss of doppler flow and/or loss of testicular volume). A moderate injury would suggest only a disruption in the *Tunica albuginea* without the other findings, a mild injury would be no sonographic breach or a suspected breach only.

Testicular salvage remains the most important outcome measure for patients with traumatic injuries to the scrotum. However, testicular atrophy is an important consequence, which needs to be considered in cases of testicular trauma. Testicular atrophy can be defined as either a significant difference in volume on subsequent ultrasound, or a clinical reduction in the volume of the testis by about 50% when compared to the contralateral side. The reabsorption of necrotic testicular tissue has been hypothesised as being the cause of testicular atrophy following testicular rupture [11]. It is unknown whether this is the singular cause or if other factors may play a causative role [12]. In our study, 30 patients developed testicular atrophy following testicular trauma, and significantly, 63% (*n* = 19) of these were managed with conservative treatment versus 37% (*n* = 11) who were managed with acute or delayed surgical treatment. It is likely that testicular ischaemia may be a result of pressure effects from oedema confined to the tunica vaginalis or haematoma development [12].

Most authors still advocate for early surgical exploration of testicular-related traumas. For this reason, caution must be taken in deciding to opt for conservative measures in any patients presenting with acute testicular trauma. Acute surgical intervention appears to be key in patients who require a surgical approach. In one series by Cass et al., delaying surgical intervention by 72 h resulted in an orchidectomy rate of 45% compared with 9% of patients who underwent acute scrotal exploration [13]. Return to function was also cited as an important factor in this case series; this can be defined as equivalent testis size and no ongoing testicular pain following trauma. Then, 40% of patients described in this case series suffered from a prolonged period of disability. However, this was not reported as an outcome measure in the majority of other studies found for this systematic review, and we feel deserves more attention in future studies on this topic.

There are a number of limitations to a study like this. There are no randomised control trials in this area, so patient cohorts included in our study lacked a control/comparison group. A major drawback of many of the papers included in the systematic review is a lack of outcome reporting. Of the 36 studies included in our systematic review, only 14 studies mentioned follow-up outcome measures such as testicular atrophy and a length of follow-up. This shows further studies with large patient cohorts are warranted before clear guidance on the long-term sequelae of each treatment modality can be definitively published. Patient population size in many of the studies is also relatively small, which may lead to sampling bias. Not all patients who underwent surgical intervention or conservative management had ultrasound imaging prior to the selection of their treatment modality, which could represent a form of selection bias in these studies.

What is clear from this review is the lack of robust evidence or a clear and structured consensus on how best to manage paediatric patients presenting with blunt scrotal trauma. Inadequacies both in study design, reporting of outcomes and standardisation in the follow-up of these patients, all contribute to the lack of good quality evidence in this area. The lack of high-quality studies reporting paediatric blunt scrotal trauma meant we were unable to identify the ages of patients presenting with this issue, and given that only 14 articles mentioned the follow-up of these patients, it resulted in a lack of data in patient outcomes based on the type of management they received.

## Conclusion

Testicular trauma in paediatric patients is infrequently reported, and currently, the quality of data and reporting of data in the literature at present is poor. All patients with scrotal trauma should be assessed with this injury in mind. Physical examination alone is not always a reliable method in diagnosing paediatric patients with a testicular rupture. There remains no clear consensus on surgical exploration of these patients versus conservative management, and at present, the decision is made based on surgeons’ preference. Although the numbers in our study are low, they would suggest that early surgery is better than delayed surgery or conservative management of testicular injuries. For those patients with testicular injury as a result of sporting injuries, surgically managed patients fared better than conservatively managed patients with regards to the development of testicular atrophy. A possible solution to this is a large, multi-institutional prospective study under the auspices of a major urology society such as the European Society for Paediatric Urology (ESPU) for example.

## Appendix 2

Table 2

**Table 2:** Criteria for the Newcastle-Ottawa Scale regarding star allocation to assess quality of studies (out of a total of seven stars)

Criteria	Acceptable (star awarded):	Unacceptable (star not awarded):
Representativeness of exposed cohort	Population-based	Hospital-based
Selection of non-exposed cohort	Same setting as exposed cohort	Different setting from exposed cohort
Ascertainment of exposure	Secure records or directly measured	Self-reported information
Comparability	Excluded or adjusted for prior outcome in analysis	No exclusion of prior outcomes
	Adjusted for age, race, and other co-morbidities	Did not adjust for age, race, and other co-morbidities
Outcome of interest	Secure records or directly measured	Self-reported information
Adequacy of follow-up	Adjusted for missing data or follow-up > 1 month	No statement regarding missing data. No follow-up after birth
